# Clinical, Biochemical, and Radiological Presentation of RT-PCR-Positive vs RT-PCR-Negative SARS-CoV-2 Pneumonia Requiring ICU Care: An Observational, Cross-Sectional, Single-Center Study in Kalaburagi, Kalyana Karnataka

**DOI:** 10.7759/cureus.31493

**Published:** 2022-11-14

**Authors:** Swaraj Waddankeri, Kshitij Arora, Nitin Mallasure, Bharat Konin, Basavaraj G Mangshetty

**Affiliations:** 1 Internal Medicine, Mahadevappa Rampure Medical College, Kalaburagi, IND

**Keywords:** rt-pcr (real-time - reverse transcription polymerase chain reaction), sars-cov-2 (severe acute respiratory syndrome coronavirus-2), respiratory infection, ct thorax, rtpcr-sars-cov-2, covid-19 outbreak, severe acute respiratory syndrome coronavirus 2, covid-19 india, ct (computed tomography) imaging, severe acute respiratory infection

## Abstract

Introduction

Studies have reported similar clinical, biochemical, and radiological features between real-time polymerase chain reaction (RT-PCR)-positive and RT-PCR-negative patients. Therefore, the present study aims to assess differences in RT-PCR-positive versus RT-PCR-negative patients' characteristics.

Methods

We prospectively included 70 consecutive patients with typical coronavirus disease 2019 (COVID-19)-like clinical features who were either RT-PCR-positive or negative, requiring admission to the intensive care unit. The patients were classified into positive and negative RT-PCR groups and evaluated for clinical features, comorbidities, laboratory findings, and radiologic features.

Results

Fifty-seven point one percent (57.1%; 40/70) were RT-PCR positive, and 42.9% (30/70) were RT-PCR negative patients. The respiratory rate was higher among negative patients (P = 0.02), whereas the mean duration of fever was longer (3.34 vs 2.5; P = 0.022) among positive patients. At presentation, RT-PCR-negative patients had lower saturation of peripheral oxygen (SpO2) (near significant P = 0.058). Evaluation of co-morbidities revealed no differences. The neutrophil/lymphocyte ratio (NLR) (4.57 vs 6.52; P = 0.048), C-reactive protein (CRP) (9.97 vs 22.7; P = 0.007), and serum ferritin (158 vs 248.52; P = 0.010) were higher in patients who tested negative for RT-PCR. Thrombocytopenia (2.42 vs 1.76; P = 0.009), D-dimer levels (408.91 vs 123.06; P = 0.03), and interleukin (IL-6) levels (219.3 vs 80.81; P = 0.04) were significantly elevated among RT-PCR positive patients. The percentage of lung involvement in negative cases was 42.29+/-22.78 vs 36.21+/-21.8 in positive cases (P=0.23). The CT severity score was similar in both cohorts.

Conclusion

Both RT-PCR-positive and negative patients have similar clinical, biochemical, and radiological features. Considering that we are amidst a pandemic, it is advisable to have a similar approach irrespective of the RT-PCR report and triage and isolate accordingly. We recommend an RT-PCR-negative intensive care unit (ICU) ward and that the treating physician take a call on the management with a holistic approach driven clinically by the laboratory findings and helped by radiological findings. Stressing only on the RT-PCR report for management can be counterproductive.

## Introduction

Coronavirus disease 2019 (COVID-19) is a highly contagious disease caused by severe acute respiratory syndrome coronavirus 2 (SARS-CoV-2), which has engulfed the globe [1,2]. On imaging, patients infected with SARS-CoV-2 present with fever, cough, dyspnea, muscle aches, and bilateral pneumonia [3,4]. Real-time polymerase chain reaction (RT-PCR) is the most commonly used method for detecting SARS-CoV-2 [5] though there are concerns about its sensitivity [6] and the time it takes to obtain results.

It was observed that while initial RT-PCR reports of patients suspected of COVID-19 symptoms were adverse, their initial chest computed tomography (CT) revealed typical findings [3,7] of bilateral, multi-lobar ground glass opacities and consolidation, especially in the peripheral lung zones [8,9]. The throat/nasal swab is recommended to be repeated among patients with chest CT findings suggestive of COVID-19, therefore indicating a higher sensitivity for chest CT detection of COVID-19. In addition, it is proposed that chest CT can reduce the false-negative diagnosis of RT-PCR in the early stages of the disease, at initial presentation [10,11]. Though RT-PCR takes only a few hours, prevailing conditions and increased sample load can delay results, thereby causing a delay in decision-making on isolation and appropriate treatment strategies.

Initially, research focused on patients with confirmed COVID-19 pneumonia [12,13]. However, it gradually shifted its focus to patients with or without COVID-19 pneumonia, which was often associated with a smaller sample [14], a low rate of confirmed COVID-19 patients [15], or no radiological imaging [16], all of which were contributing to an appropriate outcome in patient management. In addition, research documents the benefit of using remdesivir early in COVID-19 infection [17]. Nevertheless, with the increasing burden of COVID-19 cases on healthcare professionals, greater emphasis is provided to identifying critical differences between RT-PCR positive and RT-PCR negative patients that can provide clinicians with an indicator that can assist them in deciding further courses of treatment.

Confirming COVID-19 pneumonia among patients reporting to an already overloaded healthcare setting is essential for effective management since non-confirmed patients suspected of COVID-19 pneumonia take up a decent proportion of healthcare resources. It is precisely for this reason that the rapid identification of patients with COVID-19 is imperative since an initial false-negative result could delay treatment and increase the risk of viral transmission. The present study aimed to describe and define the clinical, biochemical, and radiological findings of patients with positive and negative RT-PCR but with clinical features suggestive of COVID-19.

## Materials and methods

A single-center, cross-sectional observational study was conducted from July 16, 2020, to August 28, 2020, at a dedicated COVID hospital affiliated with Mahadevappa Rampure Medical College, Kalaburagi, Kalyana Karnataka, in South India.

We prospectively included 70 patients who fulfilled the inclusion and exclusion criteria. Patients above 18 years of age and below 80 years of age presenting with typical COVID-19-like clinical features, who were either RT-PCR positive or negative, requiring admission to the intensive care unit (ICU), defined as hypoxia (oxygen saturation (SpO2) < 94% on room air), high-resolution CT showing lesions of COVID-19 (ground glass opacities), and raised inflammatory markers (D-dimer, ferritin, lactate dehydrogenase (LDH), interleukin 6 (IL-6)), or requiring admission. Any of these parameters, in isolation or combination, were included for evaluation. Asymptomatic patients aged < 18 years and > 80 years with normal, high-resolution CT thorax, pregnant and nursing mothers, underlying chronic or newly diagnosed liver and renal dysfunction, and pre-existing malignancy were excluded from the evaluation. Asymptomatic patients aged < 18 and > 80 years with normal high-resolution CT thorax, pregnant and nursing mothers, underlying chronic or newly diagnosed liver and renal dysfunction, and pre-existing malignancy were excluded from evaluation. The study was done in accordance with the Declaration of Helsinki. Written informed consent was obtained from each patient or the patient's legally authorized representative if the patient could not provide consent.

The data were entered in accordance with a preset proforma deliberately made for the assessment of COVID-19 pneumonia. In addition, laboratory data, radiological imaging (CT), and RT-PCR for SARS-CoV-2 were recorded.

The high-resolution computerized tomography of the chest was done on a Philips 16-slice CT machine (Philips, Amsterdam, Netherlands). The study blinded radiologist (for RT-PCR status and lab results) opined with respect to the CT findings based on the observed lesions. Post the radiological report, the patients were classified as having RT-PCR-positive pneumonia and RT-PCR-negative pneumonia.

The CT consisted of contiguous axial sections of a thickness of 5 mm of the thorax in the craniocaudal direction. Reconstruction was done with a slice thickness of 1.25 mm. All images are viewed in a range of lung and mediastinal window settings. A semi-quantitative scoring system was used to estimate lung involvement quantitatively based on the area involved. Each of the five lobes was visually scored from 0 to 5. As 0 = no involvement, 1 = 5% involvement, 2 = 25% involvement, 3 = 26-49% involvement, 4 = 50-75% involvement, and 5 = > 75% involvement. The total CT score is the sum of lung involvement (5 lobes, score 1-5 for each lobe, range 0: none to 25: maximum) [18].

COVID-19 typical features were ground glass opacities (GGOs) with or without "crazy paving" and consolidations with peripheral emphasis. In addition, pneumonic findings in CT scans were evaluated per lung lobe regarding the presence of ground glass opacities and consolidations.

Statistical analysis

The data were entered in Microsoft Excel (Microsoft Office 2013, Microsoft Corp. USA), explored using Python (https://www.python.org/), and analyzed using SPSS (SPSS version 20, IBM Corp., Armonk, NY)) and R software (R Core Team (2021)). The chi-square and Fisher tests were used for qualitative data, and an unpaired t-test was applied for quantitative data. The level of significance was set at P ≤ 0.05.

Institutional approval

Consent was obtained from all participants in this study. The Institutional Ethics Committee M.R Medical College, Kalaburagi, issued approval ECR/889/Inst./2017. The Institutional Ethics Committee M.R Medical College, Kalaburagi, met and scrutinized the research project, "Clinical, Biochemical and Radiological presentation of RT-PCR positive vs RT-PCR Negative SARS-CoV-2 Pneumonia requiring ICU care: An Observational, Cross-sectional Single Center Study in Kalaburagi, Kalyana Karnataka" from ethical clearance point of view. After scrutiny, the original version of the research project has been accorded ethical clearance.

## Results

A total of 70 patients were enrolled in the study. It was found that 57.1% (40/70) were RT-PCR positive (with a mean age of 49.41 ± 15.75 years) and 42.9% (30/70) were RT-PCR negative (with a mean age of 53.26 ± 16.34 years). Males constituted 77.1%, and 24.3% of overall study participants were 51-60 years old. There was no significant difference in the distribution of RT-PCR findings with reference to age (P = 0.41) and gender (P = 0.32) (Table 1).

**Table 1:** Distribution of study participants according to age and gender Level of significance at P ≤ 0.05 NS–Not Significant using the chi-square test (χ2 – 1.14; P = 0.413) (NS)–Not Significant using the unpaired t-test (t – 1.02; P = 0.321)

Age in years	Positive cases		Negative cases		Total	P value
	Male	Female	Male	Female		
	No. (%)	No. (%)	No. (%)	No. (%)	No. (%)	
≤ 40	10	0	7	1	18 (25.7%)	P = 0.413 NS
41-50	6	5	4	1	16 (22.8%)	
51-60	7	2	6	2	17 (24.3%)	
61-70	4	3	3	1	11 (15.7%)	
≥71	2	1	5	0	8 (11.5%)	
Total	29	11	25	5	70 (100.0%)	
Mean ± SD	49.41 ± 15.75		53.26 ± 16.34		53.26 ± 16.06	P = 0.321 (NS)

Clinical characteristics

**Figure 1:**
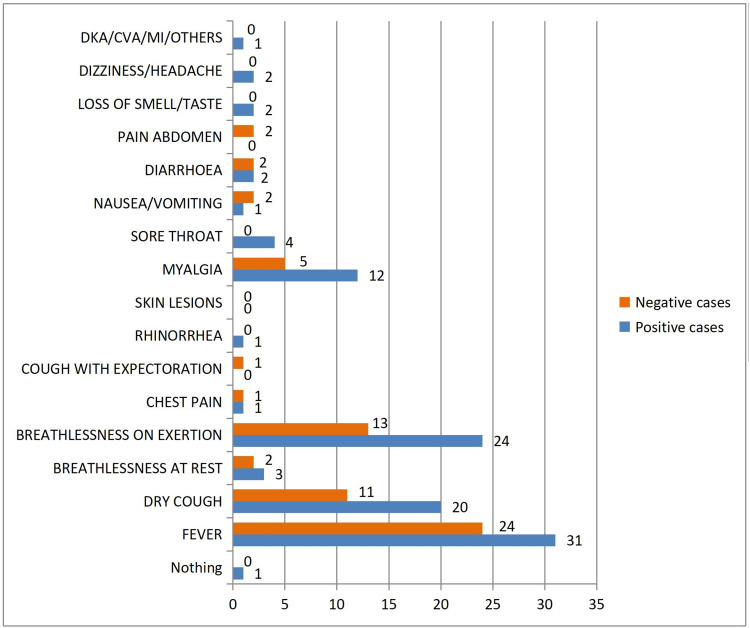
Distribution of cases according to presenting complaints Comparison of presenting complaints was not significant between RT-PCR-positive and RT-PCR-negative patients (P = 0.352) RT-PCR–Real-Time Polymerase Chain Reaction

Comparing the symptoms in RT-PCR-positive and RT-PCR-negative patients showed no significant difference. Though not significant, fever (78.6%), dyspnea on exertion (52.9%), and dry cough (44.3%) were the main presenting symptoms among both RT-PCR-positive and RT-PCR-negative patients (Figure 1).

Statistical analysis of initial vital signs showed that the patients' mean systolic and diastolic pressure were similar between RT-PCR-positive and RT-PCR-negative patients (P > 0.05). At the same time, the respiratory rate was significantly higher among RT-PCR-negative patients (P = 0.02). SpO2 levels were lower in RT-PCR-negative patients (P = 0.058). There were no significant changes in electrocardiogram (ECG) between RT-PCR-positive and RT-PCR-negative patients. Simultaneously, negative cases were more hypoxic than positive cases though the difference was not statistically significant (SpO2 93.95% 6.08 (positive) vs 90.97% 6.80 (negative) P = 0.058) (Table 2 and Table 3).

**Table 2:** Comparison of duration of illness of presenting complaints among RT-PCR-positive and negative cases SD–Standard Deviation; a–data not sufficient; DKA–Diabetic Ketoacidosis; CVA–Cerebrovascular Accident; MI–Myocardial Infarction; RT-PCR–Real-Time Polymerase Chain Reaction Level of significance at P ≤ 0.05 *statistically significant using the unpaired t-test

Presenting complaints	Positive cases: Duration of illness	Negative cases: Duration of illness	P value
	Mean ± SD	Mean ± SD	
Fever	3.34 ± 1.53	2.50 ± 1.10	P = 0.022*
Dry cough	3.04 ± 1.21	3.63 ± 1.12	P = 0.187
Breathlessness at rest	-	-	-
Breathlessness on exertion	2.13 ± 1.24	2.77 ± 1.26	P = 0.116
Chest pain^a^	-	-	-
Cough with expectoration^a^	-	-	-
Rhinorrhea^a^	-	-	-
Skin lesions^a^	-	-	-
Myalgia	3.69 ± 1.10	3.57 ± 1.32	P = 0.695
Sore throat^a^	-	-	-
Nausea/vomiting	2.3 ± 0.73	2.5 ± 0.84	P = 0.737
Diarrhea^a^	-	-	-
Pain abdomen^a^	-	-	-
Loss of smell / taste^a^	-	-	-
Dizziness / headache^a^	-	-	-
DKA / CVA / MI / others^a^	-	-	-

**Table 3:** SpO2 and respiratory rate difference between RT-PCR-positive and negative patients SD–Standard Deviation; SpO2–Saturation of Peripheral Oxygen; RT-PCR–Real-Time Polymerase Chain Reaction *statistically significant at P < 0.05 using the chi-square test

		Positive	Negative	P value
Respiratory Rate	Normal	28 (70%)	13 (43.3%)	P = 0.028*
	Tachyapnoea	12 (30%)	17 (56.7%)	
SpO_2_	Mean ± SD	93.95 ± 6.08	90.97 ± 6.80	P = 0.058

Radiological characteristics

Chest CT examination revealed significant involvement of the left upper lobe (P = 0.01) and right upper lobe (P = 0.007) in RT-PCR-positive patients. In addition, ground glass opacities were significantly prominent among RT-PCR-positive patients that involved the left upper lobe (P = 0.02) and right upper lobe (P = 0.007), respectively (Table 4).

**Table 4:** Comparison of investigation between RT-PCR-positive and negative cases ECG–Electrocardiogram; HRCT–High Resolution Computed Tomography; IHD–Ischaemic Heart Disease; RT-PCR–Real-Time Polymerase Chain Reaction Level of significance at P ≤ 0.05; Statistically significant at *P ≤ 0.05 and **P ≤ 0.01 using the chi-square test

Investigations		Positive cases (n = 40)	Negative cases (n = 30)	P value
ECG	Normal Sinus Rhythm	16 (40.0%)	11 (36.7%)	P = 0.785
	Sinus Tachycardia	22 (55.0%)	19 (63.3)	P = 0.841
	IHD	1 (2.5%)	0 (0.0%)	--
	Q waves in Inferior leads	1 (2.5%)	0 (0.0%)	--
HRCT Thorax	Normal	4 (10.0%)	5 (16.7%)	P = 0.342
	Left Lower Lobe	29 (72.5%)	21 (70.0%)	P = 0.451
	Left Middle Lobe	5 (12.5%)	1 (3.3%)	P = 0.131
	Left Upper Lobe	26 (65.0%)	13 (43.3%)	P = 0.010**
	Right Lower Lobe	28(70.0%)	21 (70.0%)	P = 0.85
	Right Upper Lobe	29 (72.5%)	13 (43.3%)	P = 0.007**
	Pleural Effusion	0 (0.0%)	2 (6.7%)	---
Ground Glass Opacities	No	4 (10.0%)	7 (23.3%)	P = 0.107
	Left Lower Lobe	28 (70.0%)	20 (66.7%)	P = 0.316
	Left Middle Lobe	5 (12.5%)	1 (3.3%)	P = 0.131
	Left Upper Lobe	25 (62.5%)	13 (43.3%)	P = 0.023*
	Right Lower Lobe	29 (72.5%)	21 (70.0%)	P = 0.451
	Right Upper Lobe	29 (72.5%)	13 (43.3%)	P = 0.007**

Both RT-PCR positive and RT-PCR negative patients had similar CT scores (10.91 vs. 10.04; P = 0.56) and percentage of lung involvement. (43.2 vs. 36.21; P = 0.239) (Table 5).

**Table 5:** Comparison of investigative findings between positive and negative cases using chest computed tomography (CT) CT–Computed Tomography Level of significance at P ≤ 0.05; Statistically significant at *P ≤ 0.05 and **P ≤ 0.01 using the chi-square test

Variables		Positive cases (n = 40)	Negative Cases (n=30)	P value
Consolidation	Yes				3(7.5%)	4 (13.3%)	P = 0.835
	No	37 (92.5%)	26 (86.7%)				
Crazy paving pattern	Yes	1 (2.5%)	1 (3.3%)	P = 0.943
	No	39 (97.5%)	29 (96.7%)	
Vascular dilatation	Yes	1 (2.5%)	0 (0.0%)	P = 0.973
	No	39 (97.5%)	30 (100.0%)	
Bronchiectasis	Yes	1 (2.5%)	0 (0.0%)	P = 0.985
	No	39 (97.5%)	30 (100.0%)	
Sub-Pleural Bands	Yes	3 (7.5%)	1 (3.3%)	P = 0.912
	No	37 (92.5%)	29 (96.7%)	
Cavitations	Yes	0 (0.0%)	0 (0.0%)	P = 1.00
	No	40 (100.0%)	30 (100.0%)	
Pleural Effusion	Yes	1 (2.5%)	3 (10.0%)	P = 0.912
	No	39 (97.5%)	27 (90.0%)	
Nodular Opacities	Yes	0 (0.0%)	0 (0.0%)	P = 1.00
	No	40 (100.0%)	30 (100.0%)	
Pericardial effusion	Yes	0 (0.0%)	0 (0.0%)	P = 1.00
	No	40 (100.0%)	30 (100.0%)	
Lymphadenopathy	Yes	14 (35.0%)	13 (43.3%)	P = 0.837
	No	26 (65.0%)	17 (56.7%)	
Percentage of involvement	--	43.29 ± 22.78	36.21 ± 21.80	P = 0.239
CT severity score	--	10.91 ± 5.87	10.04 ± 5.37	P = 0.567

Laboratory parameters

**Figure 2:**
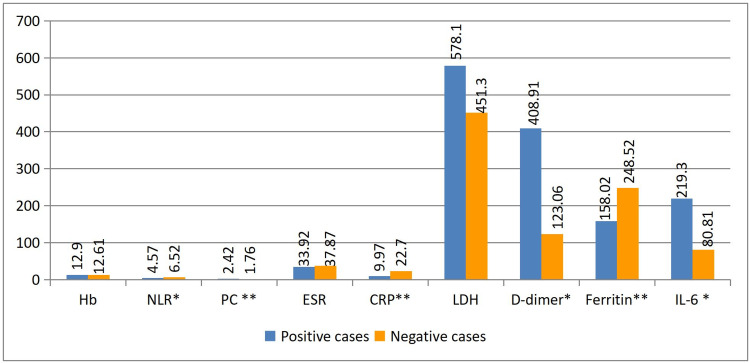
Comparison of biochemical investigations between positive and negative cases Hb–Hemoglobin (g/dL); NLR–Neutrophil/Lymphocyte; PC–Platelet Count; ESR–Erythrocyte Sedimentation Rate (mm/h); CRP–C-Reactive Protein; LDH–Lactate Dehydrogenase (U/L); D–Dimer (ng/mL); Ferrititn (ng/mL); IL-6 Interleukin 6 Statistically significant at *P ≤ 0.05 and **P < 0.01 using the unpaired t-test

**Figure 3:**
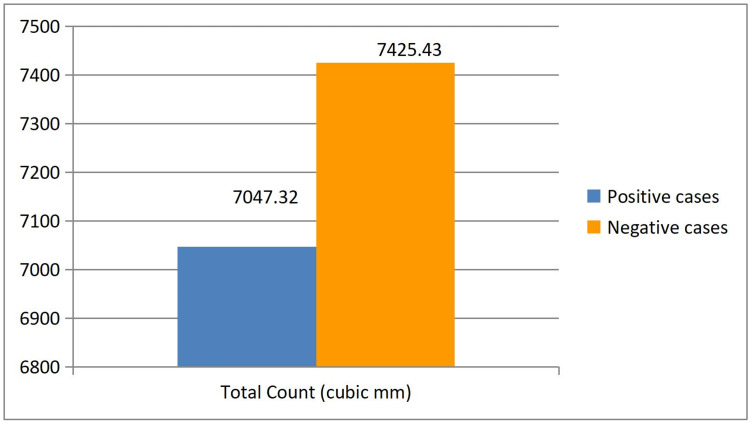
Comparison of total count between RT-PCR positive and RT-PCR negative patients RT-PCR–Real-Time Polymerase Chain Reaction

Figure 2 and Figure 3 compare the laboratory parameters of patients with positive and negative RT-PCR results. Most of the parameters were similar except for the neutrophil/lymphocyte ratio (NLR) (4.57 vs. 6.52; P = 0.048), C-reactive protein (CRP) (9.97 vs. 22.7; P = 0.007), and serum ferritin (158 vs. 248.52; P = 0.010), which was found to be significantly higher among RT-PCR-negative patients.

In RT-PCR-positive patients, thrombocytopenia (2.42 vs. 1.76; P = 0.009), D-dimer levels (408.91 vs. 123.06; P = 0.03), and interleukin (IL-6-2) levels (219.3 vs. 80.81; P = 0.04) showed statistical significance.

## Discussion

The current data highlight the differences and similarities in clinical features, laboratory values, and radiological findings of positive and negative patients. Our study was able to highlight certain features that were prominent among RT-PCR-positive patients. We observed that there was no significant difference with respect to age and gender in both groups. Though not significant, we also observed that fever, breathlessness on exertion, and dry cough were the most common presenting symptoms among RT-PCR-positive patients, similar to studies conducted in different settings [19,20]. Interestingly, tachypnea was significantly more common among RT-PCR-negative patients. It is known that respiratory rate is a standard screening tool to identify lower respiratory tract infections in clinical settings [21]. We can attribute this finding to either the patient's seeking medical care late or a superimposed secondary bacterial infection. Finally, we observed that SpO2 levels were lower in RT-PCR-negative patients. Though this might not fit in with the current pattern associated with COVID-19, we also need to accept the existence of non-communicable respiratory diseases and infections like tuberculosis that might affect patients amidst the ongoing pandemic.

Several studies have revealed typical laboratory characteristics in COVID-19 patients, such as elevated levels of C-reactive protein (CRP) and lactate dehydrogenase (LDH), as well as leukocytopenia [22-24]. Though some studies did not find any significant difference between positive and negative RT-PCR patients for most biochemical parameters [25], we observed a significant increase in the N/L ratio, CRP, and ferritin levels among RT-PCR-negative patients, which are hallmarks of inflammation. A likely explanation would be the presence of a severe respiratory system infection other than COVID-19 and a false positive report.

We also observed thrombocytopenia and elevated D-dimer and IL-6 levels among RT-PCR-positive patients. Though both D-dimer and IL-6 were higher in both RT-PCR positive and negative patients, it was significantly more among RT-PCR positive patients. Elevated D-dimer indicates an underlying coagulopathy, with patients having an increased tendency to develop vascular complications. The elevated IL-6 in our study was contrary to a previous study where there was no significant difference in IL-6 levels between positive and negative patients [26]. Positive patients may have a severe illness because higher IL-6 levels have been linked to severe illness [27].

Interestingly, we observed that though D-dimer levels were higher among positive patients, the CRP levels were significantly lower among the same group of patients, indicating that inflammation may not be the only reason responsible for the coagulation system activation. An earlier study investigating viral pathogenesis identified a novel host pathway involved in SARS progression [28]. It suggests that dysregulation of the urokinase pathway during SARS-coronavirus infection contributes to more severe lung pathology. In the present study, we also observed that compared to positive patients, negative patients had elevated total white blood cell count and Erythrocyte Sedimentation Rate (ESR), which was not significant.

The need for hospitalization and subsequent management of COVID-19 patients mainly depends on RT-PCR results. However, some studies highlight the lack of sensitivity of RTPCR and have identified COVID-19 patients based on the findings of chest CT, despite a negative RT-PCR report. In the present study, radiological findings were similar in both groups, with no significant difference in CT score and proportion of lung involvement. We found that both RT-PCR positive and negative patients showed ground glass opacities (typical of COVID-19) [29,30] that were bilateral and significantly involved the left upper lobe and right upper lobe of the lung. In addition, CT findings, such as crazy-paving patterns, vascular dilation, sub-pleural bands, pleural effusions, and others, were similar in both positive and negative RT-PCR patients.

The type of lung lesions on chest CT in RT-PCR-positive patients can be used as an imaging guideline in emergency departments to triage the patients. Although there was a ground glass appearance in other lobes of the lung in negative patients, it was not significantly greater than in positive patients. We must emphasize that, given the laboratory findings, clinical symptoms, and CT findings, we cannot categorically rule out the diagnosis of COVID-19 among RT-PCR-negative patients. We were curious if RT-PCR-negative patients (who exhibited typical CT findings) had come into contact with any COVID-19-positive patients in the past. Therefore, we can conclude that these patients were highly suspicious of COVID-19. On a positive note, the administration of newer drugs like Remdesivir, along with standard protocol, has proven to be beneficial, with a significant improvement in clinical and laboratory findings [17].

Our study has a few limitations. First, though our objective was to present the clinical, radiological, and biochemical findings of RT-PCR positive and negative patients, we did not compare the diagnostic performance between CT and RT-PCR among patients who were initially negative yet had ground glass opacities. Second, the sample size needed to be more significant. Third, we should have attempted to correlate different biomarkers that might have given us a detailed picture of the variation of significant biomarkers during the progression of the disease.

## Conclusions

It can be concluded that both RT-PCR-positive and negative patients had almost similar clinical, biochemical, and radiological features. RT-PCR-positive patients had ground glass opacities limited to the upper lobes of both their lungs. Thrombocytopenia elevated D-dimer, and IL-6 were found among RT-PCR patients, whereas CRP, NLR, and ferritin were found among RT-PCR-negative patients. We recommend an RT-PCR-negative ICU ward and that the treating physician take a call on the management with a holistic approach that is clinically aided by the laboratory findings and helped by radiological findings. Stressing only on the RT-PCR report for management can be counterproductive.
